# Potential utility of physical function measures to improve the risk prediction of functional disability in community-dwelling older Japanese adults: a prospective study

**DOI:** 10.1186/s12877-021-02415-3

**Published:** 2021-09-01

**Authors:** Tao Chen, Takanori Honda, Sanmei Chen, Hiro Kishimoto, Shuzo Kumagai, Kenji Narazaki

**Affiliations:** 1 Sport and Health Research Center, Department of Physical Education, Tongji University, 1239 Siping Road, 200092 Shanghai, China; 2 Department of Epidemiology and Public Health, Graduate School of Medical Sciences, Kyushu University, 3-1-1 Maidashi, Higashi-ku, 812-8582 Fukuoka, Japan; 3 Department of Global Health Nursing, Graduate School of Biomedical and Health Sciences, Hiroshima University, 1-2-3 Kasumi, Minami Ward, 734-8553 Hiroshima, Japan; 4 Faculty of Arts and Science, Kyushu University, 744 Motooka Nishi-ku, 819-0395 Fukuoka, Japan; 5 Institute of Convergence Bio-Health, Dong-A University, 37 Nakdong-daero 550 beon-gil, Hadan-dong, Saha-gu, 49-315 Busan, South Korea; 6 Kumagai Institute of Health Policy, 4-47-1 Hiratadai, 816-0812 Kasuga-shi, Fukuoka, Japan; 7 Center for Liberal Arts, Fukuoka Institute of Technology, 3-30-1 Wajiro- higashi, Higashi-ku, 811-0295 Fukuoka, Japan

**Keywords:** Physical performance, Predictive value, Risk assessment, Functional limitation

## Abstract

**Background:**

While gait speed, one-leg standing balance, and handgrip strength have been shown to be independent predictors for functional disability, it is unclear whether such simple measures of physical function contribute to improved risk prediction of functional disability in older adults.

**Methods:**

A total of 1,591 adults aged ≥ 65 years and without functional disability at baseline were followed up for up to 7.9 years. Functional disability was identified using the database of Japan’s Long-term Care Insurance System. Maximum gait speed, one-leg standing time, and handgrip strength were measured at baseline. Cox proportional hazard models were used to estimate the hazard ratios (HRs) and 95 % confidence intervals (CIs) for the association of physical function and functional disability incidence. The incremental predictive value of each physical function measure for risk prediction was quantified using the difference in overall C-statistic, category-free net reclassification improvement (NRI), and integrated discrimination improvement (IDI) index.

**Results:**

During follow-up (median: 7.8 years), functional disability was identified in 384 participants. All of the physical function measures were inversely associated with the risk of functional disability, independent of potential confounding factors. The multivariable adjusted HRs (95 % CIs) for functional disability per one standard deviation increment of maximum gait speed, one-leg-standing time, and hand grip strength were 0.73 (0.65–0.83), 0.68 (0.59–0.79), and 0.72 (0.59–0.86), respectively. Incorporation of each of maximum gait speed, one-leg-stand time, and hand grip strength into a basic model with other risk factors significantly improved C-statistic from 0.770 (95 % CIs, 0.751–0.794) to 0.778 (0.759–0.803), 0.782 (0.760–0.805), and 0.775 (0.756–0.800), respectively (all p < 0.05). A model including all three measures had the highest C-statistic of 0.787 (0.765–0.810). The improvements in risk prediction were also confirmed by category-free NRI and IDI index.

**Conclusions:**

Adding any of the three measures to a basic model with other known risk factors significantly improved the prediction of functional disability and addition of all three measures provided further improvement of the prediction in older Japanese adults. These data provide robust evidence to support the practical utility of incorporating these simple physical function measures into functional disability risk prediction tools.

**Supplementary Information:**

The online version contains supplementary material available at 10.1186/s12877-021-02415-3.

## Introduction

Functional disability in older adults causes increased acute care use [1], hospitalization [2], and death [3], and places considerable burdens on social, economic, and healthcare systems [4], underscoring the need for strategies to delay the onset of functional disability. Accurate identification of individuals who are at high risk is of great importance for implementing cost-effective prevention strategies. Although several multifaceted models based on self-reported risk factors have been developed for predicting incident functional disability among older adults, the discriminatory accuracy of these models (C-statistic range: 0.66–0.79) [5–8] has only been modest [9], highlighting unexplained variation in the risk of functional disability.

Objectively measured physical functions, including gait speed, one-leg standing balance, and handgrip strength, have been shown to be strong independent predictors for future functional disability in older adults [10, 11], and have increasingly been accepted as biomarkers of aging [12, 13]. Thus, objectively measured physical functions may contribute information beyond that obtained from other traditional risk factors and thereby improve the prediction of risk for functional disability. However, few studies have examined the added predictive values of objectively measured physical functions in the context of many other known risk factors for functional disability [14]. Furthermore, most studies have examined the predictive ability using relative risk alone, which is not sufficient to clearly define the predictive utility of a marker [15].

Given the highly dynamic nature of disability (e.g., recovery rates of activities of daily living (ADLs) disability as high as 81 % within 12 months, and 65 % of disability episodes lasting only 1 or 2 months) [16], prior studies with assessment intervals of ≥ 6-month or even years [11] may underestimate the incidence of functional disability and thus lead to imprecise risk prediction. As noted in the literature [14], another common issue when studying functional disability as the outcome is loss to follow-up due to disability itself, which would underestimate the true effects [17].

To address the issues of ascertainment of functional disability and gaps in knowledge about the predictive utility of objectively measured physical functions, we linked the data from a prospective cohort study of community-dwelling older adults, including objective measures of physical function and other potential risk factors, to the Long-term Care Insurance (LTCI) system [18]. The LTCI system has been a mandatory program since it was implemented in 2000, and every adult in Japan, aged 65 years or older, is eligible for the benefits, based strictly on assessment of physical and mental disability including physicians’ standardized examination [18]. The aims of the present study were to explore whether incorporation of any of the physical function measures examined (maximum gait speed, one-leg standing balance, and handgrip strength) in a model with other common risk factors could improve prediction of functional disability, as ascertained using the LTCI system, in a nearly 8-year prospective cohort study of older Japanese adults.

## Methods

### Participants

This prospective study used data from the Sasaguri Genkimon Study (SGS), which is an ongoing, community-based prospective study in Sasaguri, a suburban town in Fukuoka, Japan, aiming to explore risk and protective factors related to long-term care needs [19]. Briefly, at the end of January 2011, a total of 4,979 Sasaguri residents aged ≥ 65-years-old, and not certified as requiring long-term care according to the LTCI system, met the SGS inclusion criteria. After excluding subjects who had died or moved out of the district (n = 66) by the onset of the study, 4,913 subjects were invited to participate and 2,629 consented. We excluded nine subjects certified as requiring LTCI before the date of their baseline assessment, 15 with self-reported medical history of dementia or Parkinson’s disease, 762 without objectively measured physical functions, and 252 with missing data regarding other risk factors. The final sample comprised 1,591 adults. Supplementary Table 1 shows the characteristics of the included participants and excluded individuals.

### Functional disability

Functional disability was identified using the nationally uniform database of the LTCI system and data were provided by the municipal government office. Certification of the LTCI system has been reported in detail elsewhere [18]. Briefly, upon the request of an elderly person or their caregiver, a trained local-government official visits the home to evaluate the applicant’s long-term care needs using a nationally standardized questionnaire on current physical and mental status, including paralysis and limitation of joint movement, movement and balance, complex movement, conditions requiring special assistance, conditions requiring assistance with ADLs/instrumental ADLs (IADLs), communication and cognition, and behavioral problems. A computer-based, standardized scoring system is used to calculate scores for physical and mental function and estimate the amount of time required for care in eight categories (grooming and bathing, eating, using the toilet, transferring, assistance with IADLs, behavioral problems, rehabilitation, and medical services). Finally, a local Nursing Care Needs Certification Board (comprising physicians, nurses, and other experts in health and social services) decides whether the older adult should be certified as requiring long-term care and assign the care needs at one of seven levels (support level, 1–2; care level, 1–5). The levels of LTCI certification have been shown to be highly correlated with the Barthel Index (Spearman’s ρ= −0.86) and moderately correlated with Mini-Mental State Examination (MMSE) scores (Spearman’s ρ= −0.42) [20]. We defined functional disability as the onset of long-term care needs at support level 1 or above [21, 22].

Participants were followed from the date of the baseline survey until one of the following: being ascertained as needing long-term care; death; loss to follow-up, because of moving out of town; or March 31, 2019, whichever came first. Information on death or moving out of the town was also provided by Sasaguri municipal government office using the resident registration system.

### Baseline physical function measures

Physical function was assessed using three objective measures: the 5-meter maximum gait speed test, the open-eyed one-leg standing test, and the handgrip test. Although usual gait speed has been more frequently used and has been shown to have additional value in prediction of ADLs disability [14], slower maximum gait speed has also been shown to be a useful predictor of future risk of disability [23, 24]. In addition, physical challenge in maximum gait speed test may uncover earlier deficits than that using usual gait speed [25]. Therefore, maximum gait speed was selected in the present study. Details of the measurements have been reported previously [26]. Briefly, in the maximum gait speed test, the participants were asked to walk along a straight 11-m lane as fast as possible in two trials, and the time (sec) for walking the 5-m distance between the 3-m and 8-m marks was measured in each trial using a digital stopwatch. The maximum gait speed (m/sec) was calculated by dividing 5 (m) by the shorter of the task times in the two trials. In the open-eyed one-leg standing test, the participants were asked to stand on their preferred leg for as long as possible (up to 120 s) while looking at a taped mark on the wall 1-m away from the toe line. This test was performed twice, and the time (sec) to failure of the task was measured in each trial using a digital stopwatch. The longer task time in the two trials was selected as the one-leg standing time (sec). The handgrip test was performed twice for each hand using a digital grip dynamometer (TKK5401; Takei Scientific Instruments, Niigata, Japan) in a standing position. Highest value of the handgrip strength test was used in analyses. Higher values indicate better physical fitness in all of the three measures. Previous studies have shown that all three measures are highly reproducible [27–29].

### Other variables

Data on age and sex were obtained from the municipality office. Living alone (yes or no), and fall experience in the previous year (yes or no) were obtained using a questionnaire. Current smoking and drinking status were assessed use questions of “do you smoke” and “do you drink” with answers of “Almost every day, Sometimes, Smoked before but not currently, Never” and “Almost every day; Sometimes, Rarely, Never”, respectively. Responses of “Almost every day” and “Sometimes” were combined and classified as current smoking or drinking. Body weight and height were measured using conventional scales, and body mass index (BMI) was calculated by dividing the body weight (kg) by height (m) squared (kg/m^2^). Multimorbidity was defined as the presence of two or more of the 13 following chronic diseases: hypertension, stroke, heart disease, diabetes mellitus, hyperlipidemia, respiratory disease, digestive disease, kidney disease, osteoarthritis or rheumatism, traumatic fracture, cancer, ear disease, and eye disease. The presence of those chronic diseases was assessed using question of “Are there any chronic diseases currently being treated or any sequelae”. Cognitive function was measured with the Japanese version of the MMSE. MMSE scores range from 0 to 30 with higher scores indicating better cognitive function. Cognitive impairment was defined as an MMSE score less than 24 [30]. Moderate-to-vigorous physical activity (MVPA) was objectively measured using a tri-axial accelerometer (Active style Pro HJA-350IT, Omron Healthcare, Kyoto, Japan) [31].

### Statistical analysis

Baseline characteristics were described using means (standard deviation [SD]), medians (interquartile range [IQR]), or proportions with Students’ t-test and chi-squared test to examine differences between men and women.

The cumulative incidence of functional disability in the overall sample was plotted using Kaplan-Meier estimates. Cox proportional hazard models were used to estimate the hazard ratios (HRs) and 95 % confidence intervals (CIs) for functional disability, according to sex-specific quartiles of each physical function measure. Model 1 adjusted for sex and age. Model 2 additionally adjusted for living alone, BMI, multimorbidity, fall experience in the past year, cognitive impairment, current smoker, current drinker, and MVPA. Model 3 also included all three physical function measures to examine whether the three measures were associated with functional disability independent of each. As we found no evidence of deviation from linearity examined using restricted cubic splines, HRs were also calculated per 1-SD increment in the respective measure. One-leg standing time was log transformed to improve the skewness for the analysis; the findings did not materially change, so results of one-leg standing time with the original scale are presented. Interactions of sex and age group (< 74 and ≥ 75 years) with respective measures were also considered, to examine any potential modifying effects of sex or age.

The incremental predictive value of each physical function measure and a combination of any two measures or all three measures for risk prediction was tested by adding each measure to a basic model with other risk factors (sex, age, living alone, BMI, multimorbidity, fall experience in the past year, cognitive impairment, smoking, drinking, and MVPA). The improvement in risk discrimination was first quantified using the difference in overall C-statistic [32]. Statistical significance was based on the standard error of the differences in the C-statistic estimated from 200 bootstrap samples, using publicly available macros [33]. We further calculated the category-free net reclassification improvement (NRI) [34] and absolute integrated discrimination improvement (IDI) index [35], which have been recommended as useful supplements to the C-statistic for evaluating increment in risk prediction accuracy offered by additional markers [36]. The probabilities of incident functional disability for each model were calculated with truncation time at 7.9 years, which was the maximum follow-up. The bootstrap method with 200 replications was also used to obtain the 95 % CIs.

All statistical analyses were conducted using SAS version 9.4 (SAS Institute Inc., Cary, NC). A significance level was set at two-sided α = 0.05.

## Results

Over a median of 7.8 years of follow-up (IQR, 6.2 to 7.8 years), 384 participants (24.1 %) developed functional disability, 108 died prior to experiencing the event, and 62 participants were lost to follow-up (4 % of the present sample). Cumulative incidence curve for the risk of functional disability was shown in Supplementary Fig. 1. Table 1 shows the baseline characteristics in the overall sample and by sex. The mean age (SD) at baseline was 73.3 (6.0) years and 39.8 % were men. Compared to women, men were less likely to be living alone, had lower rate of fall experience, were more likely to smoke and drink, and had better physical functions but less MVPA. Participants with functional disability also had shorter MVPA time and poorer physical functions.

**Table 1:** Baseline characteristics of the study population (*n* = 1,591)

Characteristic	Overall	Men (*n* = 634)	Women (*n* = 957)	*p* value*
Age, years	73.3 ± 6	73.2 ± 5.9	73.4 ± 6	0.67
Living alone, %	13.0	6.3	17.4	< 0.0001
BMI, kg/m^2^	23.2 ± 3.1	23.3 ± 2.8	23.1 ± 3.4	0.16
Multimorbidity, %	47.0	48.4	46.1	0.36
Fall experience in the past year, %	19.2	16.6	20.9	0.03
Cognitive impairment, %	5.4	6.2	4.9	0.28
Current smoker, %	7.5	15.3	2.3	< 0.0001
Current drinker, %	39.4	62.2	24.2	< 0.0001
MVPA, min/day	45 ± 34.2	41 ± 31.6	47.7 ± 35.6	< 0.0001
Maximum gait speed, m/sec	1.7 ± 0.4	1.8 ± 0.4	1.6 ± 0.4	< 0.0001
One-leg standing time, sec	42.2 (13.8–120)	48.8 (17.3–120)	38.1 (12.5–120)	0.003
Handgrip strength, kg	28.4 ± 8.2	36.1 ± 6.3	23.3 ± 4.5	< 0.0001

Note: Continuous variables are represented as mean ± standard deviation or median (IQR)

*Statistical significance based on chi-square tests or t-tests, as appropriate

*BMI* body mass index; *MVPA* moderate-vigorous physical activity

Table 2 shows the associations between physical function measures and incidence of functional disability. There were inverse associations across the quartiles of each physical function measure, with lower risk of incident functional disability in higher quartiles after adjusting for sex and age in model 1 (p for trend < 0.0001). After further adjustment in model 2, associations of all the three measures were slightly attenuated, but all remained significant. The multivariable adjusted HRs (95 % CIs) for functional disability per 1-SD increment of maximum gait speed, one-leg standing time, and hand grip strength were 0.73 (0.65–0.83), 0.68 (0.59–0.79), and 0.72 (0.59–0.86), respectively. After mutual adjustment in model 3, associations of maximum gait speed and one-leg standing time remained significant. The association of handgrip strength was attenuated to non-significant when included as continuous variable. The association of all the three measures with functional disability did not vary by sex (p > 0.20 for all interactions). As shown in Supplementary Tables 2, the associations were in the same direction in both sexes. There was also no evidence of age interaction (p > 0.05). Thus, in the subsequent analyses, overall sample was used to maximize the power.

**Table 2:** Associations between objective measures of physical function and functional disability

No. of events/subjects		Incidence rate per 1000 person-years	Model 1		Model 2		Model 3	
			HRs (95 % CIs)	*p* value	HRs (95 % CIs)	*p* value	HR (95 % CI)	*p* value
Maximum gait speed, m/sec								
Q1 (lowest)	175/395	79.7	1.00		1.00		1.00	
Q2	97/391	37.8	0.63 (0.49–0.81)	0.0003	0.66 (0.51–0.86)	0.002	0.67 (0.52–0.87)	0.003
Q3	75/404	26.5	0.56 (0.42–0.75)	< 0.0001	0.64 (0.48–0.85)	0.003	0.70 (0.52–0.94)	0.02
Q4 (highest)	37/401	12.5	0.33 (0.22–0.48)	< 0.0001	0.40 (0.27–0.59)	< 0.0001	0.49 (0.33–0.73)	0.0004
p for trend				< 0.0001		< 0.0001		0.0008
Per 1 SD increment			0.69 (0.61–0.77)	< 0.0001	0.73 (0.65–0.83)	< 0.0001	0.81 (0.71–0.92)	0.001
One-leg standing time, sec								
Q1 (lowest)	161/397	70.5	1.00		1.00		1.00	
Q2	131/398	52.3	1.05 (0.83–1.34)	0.67	1.06 (0.84–1.36)	0.62	1.16 (0.91–1.49)	0.22
Q3	55/318	24.6	0.60 (0.43–0.83)	0.002	0.67 (0.49–0.93)	0.02	0.78 (0.56–1.09)	0.14
Q4 (highest)	37/478	10.5	0.36 (0.24–0.53)	< 0.0001	0.41 (0.28–0.61)	< 0.0001	0.51 (0.34–0.77)	0.001
p for trend				< 0.0001		< 0.0001		0.001
Per 1 SD increment			0.64 (0.55–0.73)	< 0.0001	0.68 (0.59–0.79)	< 0.0001	0.74 (0.63–0.86)	< 0.0001
Handgrip strength, kg								
Q1 (lowest)	149/377	69.0	1.00		1.00		1.00	
Q2	116/359	50.5	0.94 (0.74–1.21)	0.63	1.02 (0.79–1.30)	0.89	1.13 (0.88–1.46)	0.33
Q3	71/393	26.3	0.63 (0.47–0.85)	0.0024	0.73 (0.54–0.98)	0.04	0.85 (0.62–1.15)	0.29
Q4 (highest)	48/462	14.2	0.45 (0.31–0.64)	< 0.0001	0.53 (0.37–0.76)	0.0005	0.69 (0.48–0.997)	0.048
p for trend				< 0.0001		0.0003		0.047
Per 1 SD increment			0.64 (0.53–0.76)	< 0.0001	0.72 (0.59–0.86)	0.0005	0.83 (0.68–1.01)	0.07

Note: Model 1 is adjusted for sex and age

Model 2 is adjusted for sex, age, living alone, body mass index, multimorbidity, fall experience in the past year, cognitive impairment, smoking, drinking, and moderate-to-vigorous physical activity

Model 3 is adjusted for variables in model 2 plus the other physical function measures

The sex-specific quartile cut points were: gait speed, 1.6, 1.8, and 2.1 m/sec for men, and 1.4, 1.6, and 1.9 m/sec for women; one-leg standing time, 17.3, 48.8, and 120 s for men, and 12.5, 38.1, and 120 s for women; handgrip strength, 32.0, 36.0, and 40.0 kg for men, and 20.5, 23.0, and 26.0 kg for women

*HRs* hazard ratios; *CIs* confidence intervals

Maximum gait speed, one-leg standing time, and handgrip strength improved the C-statistic of the basic model from 0.770 (95 % CIs, 0.751–0.794) to 0.778 (0.759–0.803), 0.782 (0.760–0.805), and 0.775 (0.756–0.800), respectively (all *p *< 0.05) (Table 3). An addition of any combination of two measures also significantly improved the C-statistic of the basic model (all *p* < 0.05). A model including all three measures produced the highest C-statistic (0.787), when compared to the basic model or a model including only one physical function measure (all *p* < 0.05). The category-free NRI and IDI index also confirmed the improvements in risk prediction.

**Table 3:** The improvement in functional disability risk discrimination when adding each physical function measure as continuous variables to the basic model

	C-statistic	Category-free NRI	Absolute IDI
Basic model	0.770 (0.751 to 0.794)	Reference	Reference
Basic model + maximum gait speed	0.778 (0.759 to 0.803)^*^	0.267 (0.173 to 0.368)^*^	0.015 (0.007 to 0.024)^*^
Basic model + one-leg standing time	0.782 (0.760 to 0.805)^*^	0.405 (0.293 to 0.516)^*^	0.022 (0.017 to 0.028)^*^
Basic model + handgrip strength	0.775 (0.756 to 0.800)^*^	0.129 (0.013 to 0.242)^*^	0.009 (0.004 to 0.015)^*^
Basic model + maximum gait speed + one-leg standing time	0.785 (0.764 to 0.809)^*^	0.448 (0.336 to 0.551)^*^	0.031 (0.023 to 0.041)^*^
Basic model + maximum gait speed + handgrip strength	0.781 (0.761 to 0.805)^*^	0.308 (0.196 to 0.427)^*^	0.019 (0.010 to 0.029)^*^
Basic model + one-leg standing time + handgrip strength	0.784 (0.763 to 0.807)^*^	0.378 (0.272 to 0.517)^*^	0.027 (0.021 to 0.035)^*^
Basic model + all three physical function measures	0.787 (0.765 to 0.810)^*^	0.429 (0.322 to 0.554)^*^	0.034 (0.025 to 0.045)^*^

Note: Basic model: sex, age, living alone, body mass index, multimorbidity, fall experience in the past year, cognitive impairment, smoking, drinking, and moderate-to-vigorous physical activity

*p < 0.05 for difference with the basic model

*NRI* net reclassification improvement; *IDI* integrated discrimination improvement

Similar results were observed when adding quartiles of physical function measure to the basic model (Supplementary Table 3).

## Discussion

In this nearly 8-year prospective study of community-dwelling older Japanese adults, the incorporation of each of the three physical functions (maximum gait speed, one-leg standing time, and handgrip strength) to a model with well-known risk factors improved discrimination of future functional disability and the simultaneous addition of all three measures further improved the risk prediction. These results provide strong evidence to support the practical utility of incorporating these simple physical function measures into functional disability risk prediction tools.

Our findings of associations between physical functions and risk of functional disability are generally consistent with a limited number of Japanese cohort studies of usual or maximum gait speed [24, 37, 38], one-leg standing time [39], and handgrip strength [37, 40], which assessed functional disability using the LTCI system. Although no significant association of one-leg standing time [24] or handgrip strength [39] with functional disability was reported, the relatively small sample sizes (n = 60 to 784) and short follow-up periods (5–6 years) may have limited the statistical power in these two earlier studies. A recent meta-analysis also revealed that poorer performance in gait speed, one-leg standing balance, and handgrip strength, measured at baseline, were associated with higher odds of disability in ADLs or instrumental ADLs at follow-up [10]. Our findings also confirmed the inverse association between each of these physical function measures and the risk of functional disability as ascertained using the LTCI system.

Although extensive research has shown associations between objective physical function measures and functional disability, whether such measures have practical utility has been unclear because a significant association does not necessarily translate into predictive utility [15]. A recent study using a pooled analysis of 27,200 older adults with disability defined by selected tasks (bathing or dressing, and mobility difficulty) reported that usual gait speed significantly increased the area under the receiver operator characteristic curve (C-statistic), after accounting for age, sex, BMI, prior hospitalization, and selected chronic conditions over 3 years of follow-up [14]. A key finding of the present study, which is the first to report it, is that adding each of one-leg standing time and handgrip strength, in addition to maximum gait speed, significantly improved the C-statistic of a basic model with well-known risk factors for functional disability. The NRI and IDI results in the present study also confirmed the improved risk discrimination. These findings extend prior observations on the associations of physical function measures with risk of functional disability, by providing novel evidence that incorporating each of these physical function measures has the potential to provide a meaningful improvement in identification of older adults at risk of future functional disability. One possible explanation for these findings is that objective measures of physical functions, acting as biomarker of aging [12, 13], provide informative indications of subclinical disease and underlying aging processes, which may not be captured by other traditional risk factors.

In addition, we also found incorporation of all three physical function measures further improved the risk prediction of functional disability, suggesting each physical function measure may capture specific underlying characteristics that is of additional value in improving the risk prediction of functional disability. The independent associations, particularly of maximum gait speed and one-leg standing time, observed in the present study also support that different measures of physical function may reflect distinct abilities. These findings provided the rationale of a combination use of different physical function measures in assessing the risk of functional disability. Given that the physical function measures included in the present study are all highly reproducible tests [27–29] and could be done quickly in different settings, including these simple objective measures of physical function as part of the routine geriatric assessment could be practical and useful for identification of high-risk individuals.

The strengths of the present study include the large community-based population and the use of the nationally standardized LTCI database, which allowed us to conduct a more formal time-to-disability analysis with a follow-up rate of almost 100 %. However, the present study also had several limitations. First, because an older person must contact the municipal government to have the care needs officially certified [18], some individuals with disability may have failed to report. Thus, the present study may have underestimated the incidence of functional disability and the true associations of physical function measures. Second, although a range of important risk factors were included in the analysis, unmeasured confounding or residual effects may still exist. Third, a large proportion of participants were excluded in the present study, mainly owing to missing data. Included participants were younger, had lower rates of falls and cognitive impairment, were less likely to smoke or drink, and to have poorer performance in the maximum gait speed and one-leg standing tests, although the participants had a higher rate of multimorbidity than did those excluded from the present study (Supplementary Table 1). Thus, participants in the present study could have been physically healthier than the general population, and it is possible that the observed results have underestimated the strength of the association between physical function measures and functional disability. In addition, the generalizability of the findings may also be limited because the present study was undertaken in a single Japanese town.

In summary, the present study demonstrated that maximum gait speed, one-leg standing time, and handgrip strength have a predictive value for functional disability beyond known risk factors and a combination use of the three measures further improve the risk prediction in community-dwelling, older Japanese adults. These findings highlight the predictive utility of incorporating these simple measures of physical function into the screening setting, to identify older adults who are at high risk of developing functional disability.

## Supplementary Information

**Additional file 1:**

**Additional file 2:**

## Data Availability

The datasets used and/or analyzed during the current study are available from the corresponding author on reasonable request.

## Abbreviations

ADLs: Activities of daily living
BMI: Body mass index
CIs: Confidence intervals
HRs: Hazard ratios
LTCI: Long-term Care Insurance
MMSE: Mini-Mental State Examination
MVPA: Moderate-to-vigorous physical activity
NRI: Category-free net reclassification improvement
IDI: Integrated discrimination improvement
SD: Standard deviation
IQR: Interquartile rang
SGS: Sasaguri Genkimon Study
