# Supplementary material for: Potential utility of physical function measures to improve the risk prediction of functional disability in community-dwelling older Japanese adults: a prospective study
Source: BMC Geriatr. 2021 Sep 1;21:476. doi: 10.1186/s12877-021-02415-3 (PMC8411504; doi:10.1186/s12877-021-02415-3)
Supplement: Supplementary file 1 — Additional file 1: [file 12877_2021_2415_MOESM1_ESM.docx]

**Title**: Potential utility of physical function measures to improve the risk prediction of functional disability in community-dwelling older Japanese adults: a prospective study

Tao Chen, PhD^1^, Takanori Honda, PhD^2^, Sanmei Chen, PhD^3^, Hiro Kishimoto, PhD^4^, Shuzo Kumagai, PhD ^5,6^, Kenji Narazaki, PhD^7*^

^1^Sport and Health Research Center, Department of Physical Education, Tongji University, 1239 Siping Road, Shanghai 200-092, China

^2^Department of Epidemiology and Public Health, Graduate School of Medical Sciences, Kyushu University, 3-1-1 Maidashi, Higashi-ku, Fukuoka 812-8582, Japan

^3^Department of Global Health Nursing, Graduate School of Biomedical and Health Sciences, Hiroshima University, 1-2-3 Kasumi, Minami Ward, Hiroshima 734-8553, Japan

^4^Faculty of Arts and Science, Kyushu University, 744 Motooka Nishi-ku, Fukuoka 819-0395, Japan

^5^Institute of Convergence Bio-Health, Dong-A University, 37 Nakdong-daero 550 beon-gil, Hadan-dong, Saha-gu, Busan 49-315, South Korea

^6^Kumagai Institute of Health Policy, 4-47-1 Hiratadai, Kasuga-shi, Fukuoka 816-0812, Japan

^7^Center for Liberal Arts, Fukuoka Institute of Technology, 3-30-1 Wajiro-higashi, Higashi-ku, Fukuoka 811-0295, Japan

***Address correspondence to:** Kenji Narazaki, PhD, 3-30-1 Wajiro-higashi, Higashi-ku, Fukuoka 811-0295, Japan. Email: [narazaki@fit.ac.jp](mailto:narazaki@fit.ac.jp)

Supplemental Table 1 Comparison between baseline characteristics of subjects who were included in and excluded from the present study

|  | No. of missing | Included | Excluded | p value* |
| --- | --- | --- | --- | --- |
|  |  | (n=1,591) | (n=1,038) |  |
| Men, % | 0 | 39.9 | 49.4 | <.0001 |
| Age, years | 0 | 73.3 ± 6 | 73.8 ± 6.6 | 0.04 |
| Living alone, % | 25 | 13.0 | 11.1 | 0.15 |
| BMI, kg/m^2^ | 67 | 23.2 ± 3.1 | 22.9 ± 3.3 | 0.01 |
| Multimorbidity, % | 0 | 47.0 | 42.3 | 0.02 |
| Fall experience in the past year, % | 38 | 19.2 | 24.3 | 0.00 |
| Cognitive impairment, % | 520 | 5.4 | 14.1 | <.0001 |
| Current smoker, % | 42 | 7.5 | 13.5 | <.0001 |
| Current drinker, % | 35 | 39.4 | 43.8 | 0.03 |
| MVPA, min/day | 659 | 45 ± 34.2 | 36.7 ± 36.2 | <.0001 |
| Maximum gait speed, m/sec | 705 | 1.7 ± 0.4 | 1.5 ± 0.5 | <.0001 |
| One-leg standing time, sec | 733 | 42.2 ( 13.8 - 120) | 22.7 ( 5.9 - 71) | <.0001 |
| Handgrip strength, kg | 772 | 28.4 ± 8.2 | 27.7 ± 8.9 | 0.22 |

Note: Continuous variables are represented as mean ± standard deviation or median (IQR).

*Statistical significance based on chi-square tests or t-tests, as appropriate.

BMI, body mass index; MVPA, moderate-vigorous physical activity.

Supplementary Table 2 Associations between objective measures of physical function and functional disability in men and women

|  | No. of events/subjects | Incidence rate per1000 person-years | Model 1 | |  | Model 2 | |
| --- | --- | --- | --- | --- | --- | --- | --- |
|  |  |  | HRs (95% CIs) | p value |  | HRs (95% CIs) | p value |
| Maximum gait speed, m/sec | |  |  |  |  |  |  |
| Men |  |  |  |  |  |  |  |
| Q1 (lowest) | 65/156 | 73.7 | 1.00 |  |  | 1.00 |  |
| Q2 | 32/155 | 31.0 | 0.48 (0.32 - 0.74) | 0.0008 |  | 0.46 (0.3 - 0.71) | 0.0005 |
| Q3 | 22/162 | 19.2 | 0.44 (0.27 - 0.74) | 0.0016 |  | 0.49 (0.29 - 0.82) | 0.0067 |
| Q4 (Highest) | 15/161 | 12.9 | 0.31 (0.17 - 0.57) | 0.0001 |  | 0.34 (0.19 - 0.62) | 0.0004 |
| p for trend |  |  |  | <.0001 |  |  | <.0001 |
| Per 1 SD increment |  |  | 0.68 (0.57 - 0.81) | <.0001 |  | 0.71 (0.59 - 0.85) | 0.0003 |
| Women |  |  |  |  |  |  |  |
| Q1 (lowest) | 110/239 | 83.7 | 1.00 |  |  | 1.00 |  |
| Q2 | 65/236 | 42.3 | 0.72 (0.52 - 0.98) | 0.0383 |  | 0.79 (0.57 - 1.09) | 0.148 |
| Q3 | 53/242 | 31.6 | 0.63 (0.45 - 0.9) | 0.0103 |  | 0.73 (0.51 - 1.05) | 0.089 |
| Q4 (Highest) | 22/240 | 12.3 | 0.33 (0.2 - 0.54) | <.0001 |  | 0.43 (0.26 - 0.71) | 0.0011 |
| p for trend |  |  |  | <.0001 |  |  | 0.0016 |
| Per 1 SD increment |  |  | 0.69 (0.59 - 0.81) | <.0001 |  | 0.75 (0.63 - 0.88) | 0.0005 |
| One-leg standing time, sec |  |  |  |  |  |  |  |
| Men |  |  |  |  |  |  |  |
| Q1 (lowest) | 60/158 | 65.3 | 1.00 |  |  | 1.00 |  |
| Q2 | 47/159 | 46.7 | 0.96 (0.64 - 1.42) | 0.8183 |  | 0.92 (0.62 - 1.38) | 0.6893 |
| Q3 | 11/101 | 15.2 | 0.41 (0.21 - 0.79) | 0.0082 |  | 0.48 (0.24 - 0.93) | 0.0309 |
| Q4 (Highest) | 16/216 | 10.1 | 0.34 (0.19 - 0.63) | 0.0005 |  | 0.39 (0.21 - 0.73) | 0.0029 |
| p for trend |  |  |  | <.0001 |  |  | 0.001 |
| Per 1 SD increment |  |  | 0.58 (0.45 - 0.73) | <.0001 |  | 0.62 (0.48 - 0.79) | 0.0001 |
| Women |  |  |  |  |  |  |  |
| Q1 (lowest) | 101/239 | 74.0 | 1.00 |  |  | 1.00 |  |
| Q2 | 84/239 | 56.0 | 1.11 (0.82 - 1.5) | 0.50 |  | 1.16 (0.85 - 1.57) | 0.35 |
| Q3 | 44/217 | 29.1 | 0.68 (0.47 - 0.99) | 0.046 |  | 0.74 (0.51 - 1.09) | 0.12 |
| Q4 (Highest) | 21/262 | 10.8 | 0.36 (0.21 - 0.6) | <.0001 |  | 0.4 (0.24 - 0.67) | 0.0006 |
| p for trend |  |  |  | <.0001 |  |  | 0.0006 |
| Per 1 SD increment |  |  | 0.67 (0.56 - 0.8) | <.0001 |  | 0.71 (0.59 - 0.85) | 0.0002 |
| Handgrip strength, kg |  |  |  |  |  |  |  |
| Men |  |  |  |  |  |  |  |
| Q1 (lowest) | 53/146 | 61.8 | 1.00 |  |  | 1.00 |  |
| Q2 | 41/158 | 40.2 | 0.89 (0.59 - 1.35) | 0.5900 |  | 0.97 (0.63 - 1.5) | 0.9057 |
| Q3 | 23/149 | 22.0 | 0.66 (0.39 - 1.11) | 0.1150 |  | 0.83 (0.49 - 1.42) | 0.4978 |
| Q4 (Highest) | 17/181 | 13.1 | 0.52 (0.28 - 0.94) | 0.0315 |  | 0.67 (0.36 - 1.24) | 0.2016 |
| p for trend |  |  |  | <.0001 |  |  | 0.1954 |
| Per 1 SD increment |  |  | 0.66 (0.5 - 0.86) | 0.0021 |  | 0.78 (0.58 - 1.03) | 0.0779 |
| Women |  |  |  |  |  |  |  |
| Q1 (lowest) | 96/231 | 73.8 | 1.00 |  |  | 1.00 |  |
| Q2 | 75/201 | 58.7 | 0.97 (0.72 - 1.32) | 0.85 |  | 1.04 (0.77 - 1.42) | 0.79 |
| Q3 | 48/244 | 29.0 | 0.62 (0.43 - 0.89) | 0.0091 |  | 0.69 (0.48 - 0.99) | 0.04 |
| Q4 (Highest) | 31/281 | 14.9 | 0.41 (0.27 - 0.64) | <.0001 |  | 0.49 (0.31 - 0.75) | 0.0013 |
| p for trend |  |  |  | <.0001 |  |  | 0.0006 |
| Per 1 SD increment |  |  | 0.62 (0.48 - 0.79) | <.0001 |  | 0.68 (0.53 - 0.88) | 0.0033 |

Note: Model 1 is adjusted for age.

Model 2 is adjusted for age, living alone, body mass index, multimorbidity, fall experience in the past year, cognitive impairment, smoking, drinking, and moderate-to-vigorous physical activity.

The sex-specific quartile cut points were: maximum gait speed, 1.6, 1.8, and 2.1 m/sec for men, and 1.4, 1.6, and 1.9 m/sec for women; one-leg standing time, 17.3, 48.8, and 120 sec for men, and 12.5, 38.1, and 120 sec for women; handgrip strength, 32.0, 36.0, and 40.0 kg for men, and 20.5, 23.0, and 26.0 kg for women.

HRs, hazard ratios; CIs, confidence intervals.

Supplemental Table 3 The improvement in functional disability risk discrimination when adding each physical function measure as sex-specific quartiles to the basic model

|  | C-statistic | | Category-free NRI | | Absolute IDI | |
| --- | --- | --- | --- | --- | --- | --- |
| Basic model | 0.77 (0.751 to 0.794) |  | Reference |  | Reference |  |
| Basic model + maximum gait speed | 0.781 (0.762 to 0.805)^*^ |  | 0.160 (0.052 to 0.272)^*^ | | 0.017 (0.009 to 0.025)^*^ | |
| Basic model + one-leg standing time | 0.781 (0.760 to 0.805)^*^ |  | 0.499 (0.390 to 0.612)^*^ | | 0.025 (0.020 to 0.030)^*^ | |
| Basic model + handgrip strength | 0.777 (0.759 to 0.800)^*^ |  | 0.432 (0.323 to 0.543)^*^ | | 0.011 (0.007 to 0.016)^*^ | |
| Basic model + maximum gait speed + one-leg standing time | 0.789 (0.767 to 0.811)^*^ |  | 0.454 (0.350 to 0.563)^*^ | | 0.037 (0.028 to 0.047)^*^ | |
| Basic model + maximum gait speed + handgrip strength | 0.785 (0.766 to 0.811)^*^ |  | 0.408 (0.282 to 0.514)^*^ | | 0.024 (0.016 to 0.033)^*^ | |
| Basic model + one-leg standing time + handgrip strength | 0.786 (0.766 to 0.809)^*^ |  | 0.437 (0.342 to 0.541)^*^ | | 0.031 (0.025 to 0.040)^*^ | |
| Basic model + all three physical function measures | 0.792 (0.771 to 0.814)^*^ |  | 0.447 (0.327 to 0.555) ^*^ | | 0.040 (0.031 to 0.052)^*^ | |

Note: Basic model: sex, age, living alone, body mass index, multimorbidity, fall experience in the past year, cognitive impairment, smoking, drinking, and moderate-to-vigorous physical activity.

*p<0.05 for difference with the basic model.

NRI, net reclassification improvement; IDI, integrated discrimination improvement
