# Supplementary material for: Potential utility of physical function measures to improve the risk prediction of functional disability in community-dwelling older Japanese adults: a prospective study
Source: BMC Geriatr. 2021 Sep 1;21:476. doi: 10.1186/s12877-021-02415-3 (PMC8411504; doi:10.1186/s12877-021-02415-3)

**Title**: Potential utility of physical function measures to improve the risk prediction of functional disability in community-dwelling older Japanese adults: a prospective study

Tao Chen, PhD^1^, Takanori Honda, PhD^2^, Sanmei Chen, PhD^3^, Hiro Kishimoto, PhD^4^, Shuzo Kumagai, PhD ^5,6^, Kenji Narazaki, PhD^7*^

^1^Sport and Health Research Center, Department of Physical Education, Tongji University, 1239 Siping Road, Shanghai 200-092, China

^2^Department of Epidemiology and Public Health, Graduate School of Medical Sciences, Kyushu University, 3-1-1 Maidashi, Higashi-ku, Fukuoka 812-8582, Japan

^3^Department of Global Health Nursing, Graduate School of Biomedical and Health Sciences, Hiroshima University, 1-2-3 Kasumi, Minami Ward, Hiroshima 734-8553, Japan

^4^Faculty of Arts and Science, Kyushu University, 744 Motooka Nishi-ku, Fukuoka 819-0395, Japan

^5^Institute of Convergence Bio-Health, Dong-A University, 37 Nakdong-daero 550 beon-gil, Hadan-dong, Saha-gu, Busan 49-315, South Korea

^6^Kumagai Institute of Health Policy, 4-47-1 Hiratadai, Kasuga-shi, Fukuoka 816-0812, Japan

^7^Center for Liberal Arts, Fukuoka Institute of Technology, 3-30-1 Wajiro-higashi, Higashi-ku, Fukuoka 811-0295, Japan

***Address correspondence to:** Kenji Narazaki, PhD, 3-30-1 Wajiro-higashi, Higashi-ku, Fukuoka 811-0295, Japan. Email: [narazaki@fit.ac.jp](mailto:narazaki@fit.ac.jp)

FIGURE LEGENDS

**Supplementary Figure 1.** Overall cumulative incidence for the risk of functional disability


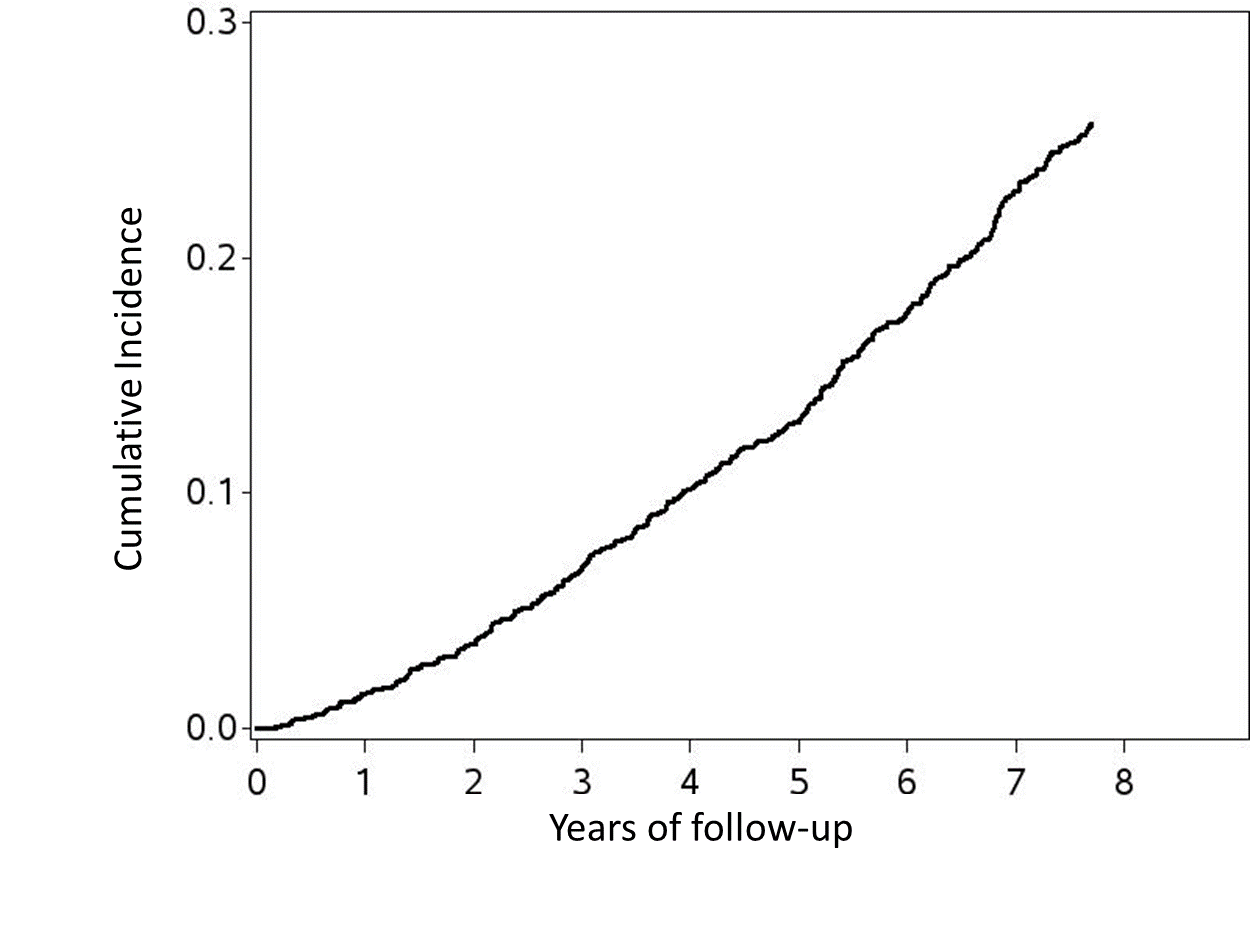

Supplement: Supplementary file 2 — Additional file 2: [file 12877_2021_2415_MOESM2_ESM.docx]
